# Radiological and Functional Outcomes Following Distal Radius Fixation With a Volar Locking Plate

**DOI:** 10.7759/cureus.91422

**Published:** 2025-09-01

**Authors:** Ahmed S Nasir, Kashif Memon, Mahmoud Mersal, Andalib Kashani, Tahreem Fatima, Serajdin Ajnin, Kuntrapaka Srininavasan

**Affiliations:** 1 Hand Surgery, Queen Elizabeth Hospital Birmingham, Birmingham, GBR; 2 Trauma and Orthopaedics, Queen Elizabeth Hospital Birmingham, Birmingham, GBR; 3 Trauma and Orthopaedics, Birmingham Heartland Hospital, Birmingham, GBR; 4 Trauma and Orthopaedics, University Hospitals Birmingham NHS Foundation Trust, Birmingham, GBR

**Keywords:** articular surface collapse, distal radius fracture, functional outcome, radiological outcome, volar locking plate

## Abstract

Introduction

With the increasing life expectancy, fractures of the distal radius are becoming more common. These are mostly treated with either closed reduction in a plaster cast or open reduction and fixation with a volar locking plate. The primary aim of our study was to assess the patient satisfaction following the volar locking plate fixation and the articular surface collapse at six weeks follow-up.

Methods

A total of 149 patients were included in the study. Measurements of radial height (RH), radial inclination (RI) and volar tilt (VT) were made pre-, intra-, and postoperatively at six weeks. The articular surface to distal screw distance (AD) was measured intraoperatively and at six weeks. Patient satisfaction was also assessed at their last follow-up.

Results

A total of 149 patients were included in this study. There were 54 (36.2%) males and 95 (63.8%) females, with a mean age of 48.9, ranging from 26 to 86 years. Preoperative mean RH, RI and VT were 8.06 mm, 14.51°, and -9.80 mm whereas the postoperative means were 12.06 mm, 22.15° and 5.94 mm, respectively. The AD intra- and postoperatively measured 5.73 mm and 4.60 mm. Of these patients, 94 (63.1%) were satisfied, 52 (34.9%) were not satisfied with the overall outcome at six weeks follow-up and three (2.0%) patients did not give any response in terms of satisfaction. The mean AD in patients satisfied with the overall outcome was 4.84 mm and 4.50 mm in those who were not satisfied.

Conclusion

Our study shows that there was a significant improvement in the distal radius radiological parameters following fixation with a volar locking plate at six weeks follow-up (P < 0.05). There was also a significant collapse of the articular surface at six weeks as shown by the decrease in AD (P < 0.05). The patients who were satisfied with the outcome had a higher average AD than those who were not satisfied.

## Introduction

Distal radius fractures represent one of the most frequently encountered fractures in orthopaedic practice, particularly in older adults. With the shift toward active aging populations and increased life expectancy, both the incidence and demand for effective treatment strategies have risen significantly [1].

Traditional conservative treatment includes closed reduction and immobilisation in a plaster cast. However, this approach has limitations, especially in cases of intra-articular involvement or unstable fracture patterns. In such scenarios, open reduction and internal fixation using a volar locking plate provides better anatomical reduction and early mobilisation [2].

Volar locking plates have become increasingly preferred due to their ability to maintain reduction in unstable and osteoporotic fractures, particularly in elderly patients [3,4]. However, concerns remain regarding long-term articular congruity and its impact on satisfaction and function.

This study aims to evaluate the radiological outcomes, including radial height (RH), radial inclination (RI), volar tilt (VT), and articular distance (AD), as the primary outcomes, and to examine their association with patient satisfaction as a secondary outcome at the routine six-week postoperative review following volar locking plate fixation of distal radius fractures. The six-week interval was chosen because it represents the routine early follow-up point in our clinical practice, when initial fracture healing is established and early collapse is most likely to be observed.

## Materials and methods

This retrospective cohort study looked at all the patients who underwent management of the distal radius between December 2022 and December 2023 across three Trust hospitals. Out of these, those who were above the age of 18 years undergoing surgical fixation with volar plate and those completing their follow-up were included. Patients undergoing conservative management with a plaster cast, K-wire fixation, aged less than 18 years or those lost to follow-up were excluded from the study. The study thus comprised 149 patients who were followed up in the outpatient clinics and assessed radiologically as well as clinically until the mean follow-up time of six weeks.

Surgical technique

All patients underwent open reduction and internal fixation using a standard volar approach. A single implant system was used across the three hospitals (Synthes® volar locking plate, DePuy Synthes, Raynham, MA, USA). Screw placement followed the manufacturer’s recommended technique, ensuring subchondral support with distal screws while avoiding intra-articular penetration. The same surgical approach and implant protocol were adopted across centres to maintain consistency.

Postoperative management and rehabilitation

Postoperatively, all patients were immobilised in a removable wrist splint for comfort and encouraged to begin early active mobilisation of the wrist and hand under physiotherapy supervision. Standard follow-up was arranged at two weeks for wound check and suture removal, and at six weeks for clinical and radiological assessment.

Patient satisfaction assessment

Patient satisfaction was assessed retrospectively by reviewing clinic letters from the six-week follow-up visits. Satisfaction was recorded as documented by the treating clinician, typically as a binary “satisfied” or “not satisfied” statement in the clinic notes. While this provided a pragmatic measure within the context of a retrospective study, it does not represent a validated patient-reported outcome tool. This limitation has been acknowledged in the Discussion.

Data collection

Standard anteroposterior and lateral wrist radiographs were obtained preoperatively, intraoperatively, and at the six-week postoperative review. Radiological parameters assessed included RH, RI, VT, and AD.

Radial height was measured as the distance (in mm) from the distal articular surface of the radius to a line perpendicular to the long axis of the radius drawn at the tip of the radial styloid.

**Figure 1:**
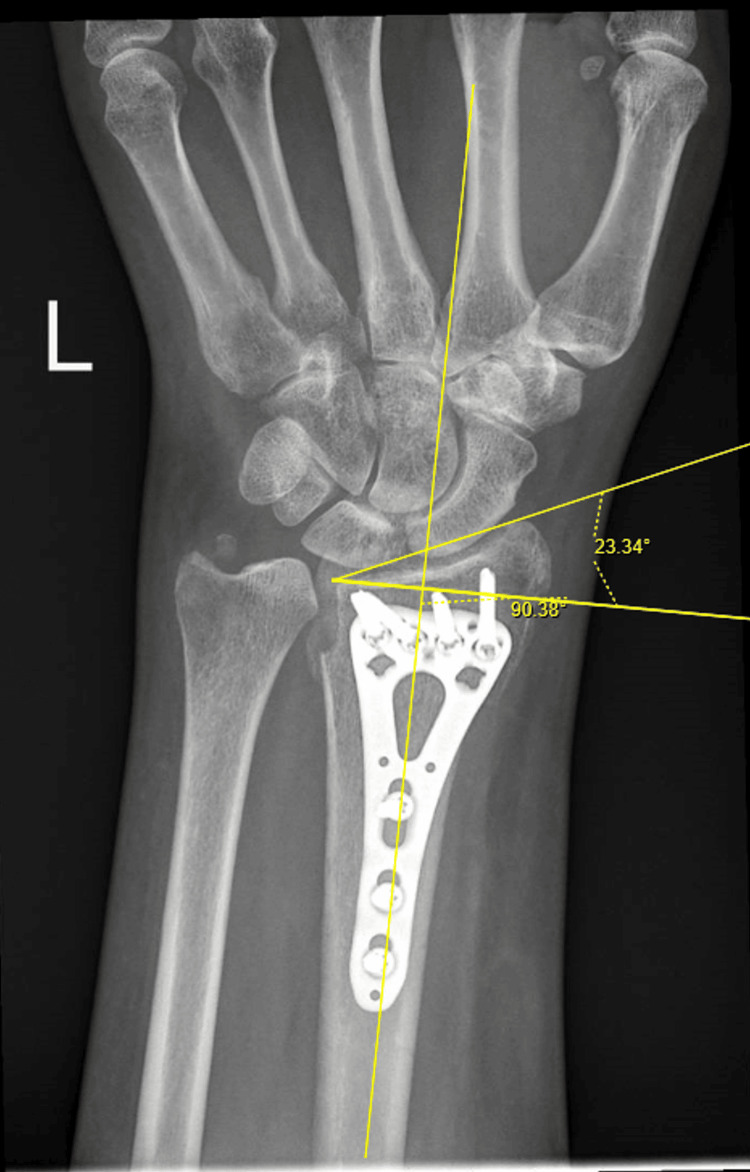
Measurement of Radial Inclination

Radial inclination (Figure 1) was measured as the angle (in degrees) between a line perpendicular to the long axis of the radius and a line connecting the tip of the radial styloid to the ulnar corner of the distal radial articular surface on the posteroanterior view.

**Figure 2:**
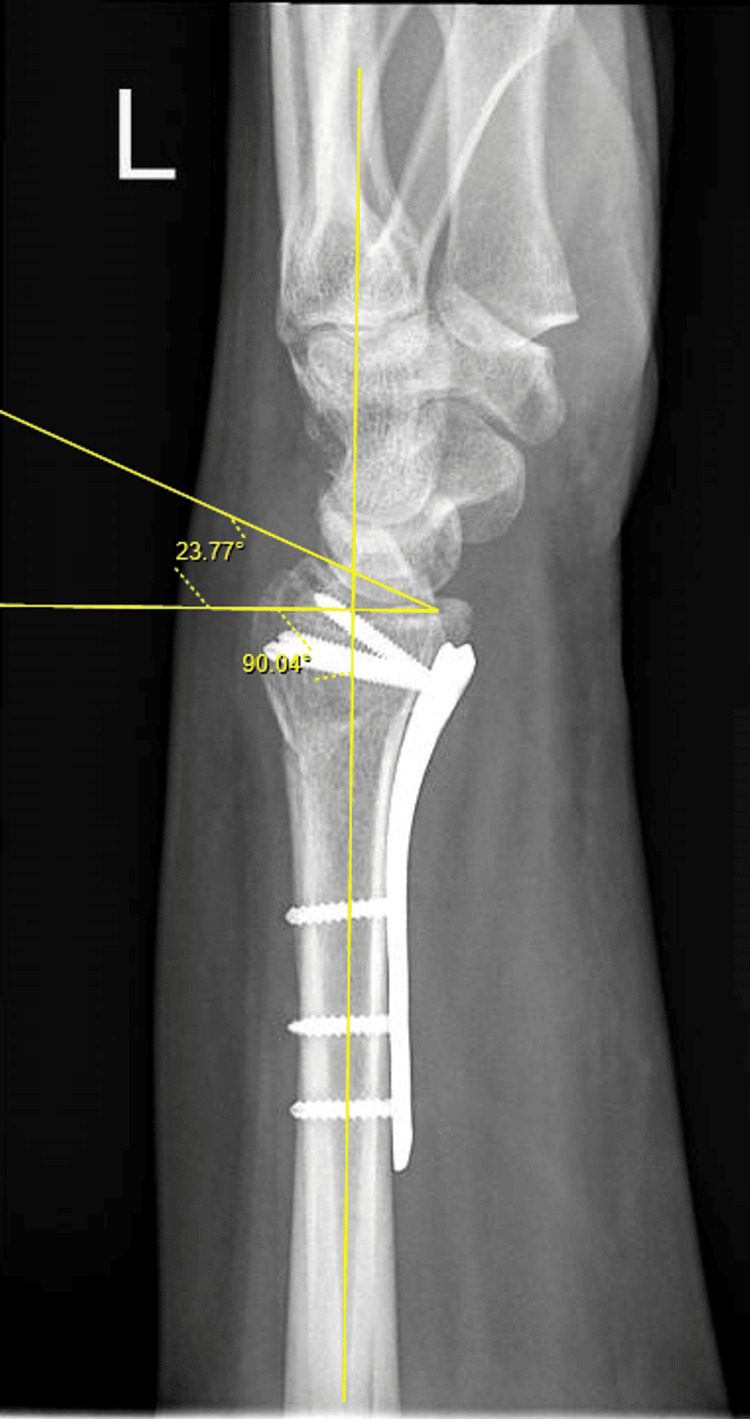
Measurement of Volar Tilt

Volar tilt (Figure 2) was measured on the lateral view as the angle (in degrees) between a line drawn along the distal radial articular surface and a line perpendicular to the longitudinal axis of the radius.

AD was defined as the shortest distance (in mm) from the distal radial articular surface to the nearest distal screw of the volar locking plate.

All measurements were performed using Carestream Vue Motion software (Carestream Health, Rochester, NY, USA) on calibrated picture archiving and communication systems (PACS) images used in the University Hospital of Birmingham Foundation Trust. Initial measurements were taken by trauma and orthopaedics doctors and independently reviewed by three consultant trauma and orthopaedic surgeons to ensure accuracy and consistency.

Patient demographics (age, sex), BMI, injury side, and satisfaction at last follow-up were recorded.

Handling of missing data

Patients who were lost to follow-up or had incomplete radiological or satisfaction data were excluded from the final analysis. No imputation was performed for missing values. Subgroup analyses were not undertaken, as the study was designed to provide a descriptive cohort-wide evaluation.

Statistical analysis

Paired t-tests were chosen as the same cohort of patients was assessed at multiple time points (preoperative, intraoperative, and six weeks), allowing direct within-subject comparisons of radiological parameters. This approach was selected to evaluate changes over time in a straightforward manner within the study cohort. A p-value of <0.05 was considered statistically significant. Analysis was conducted using Excel for Microsoft 365 MSO (Version 2503 Build 16.0.18623.20266; Microsoft, Redmond, WA, USA) t-test function.

## Results

A total of 149 patients were included in this study, comprising 54 males (36.2%) and 95 females (63.8%), with a mean age of 48.9 years (range 26-86 years) as shown in Table 1.

**Table 1:** Demographics

Total patients	N = 149
Gender	
Male	54 (36.2%)
Female	95 (63.8%)
Mean age (years)	48.9
Age range (years)	26 – 86

Radiographic parameters showed significant improvement from preoperative to postoperative assessments at six weeks follow-up (Table 2). The mean RH increased from 8.06 mm to 12.06 mm, RI improved from 14.51° to 22.15°, and VT shifted from a mean of -9.80° preoperatively to 5.94° postoperatively (P < 0.05 for all parameters). The AD decreased from a mean of 5.73 mm intraoperatively to 4.60 mm at the six-week follow-up, indicating articular surface settling or collapse (P < 0.05).

**Table 2:** Radiological Parameters

Parameter	Pre-op mean	Intra-op mean	Post-op mean	P-Value
Radial Height (mm)	8.06	14.87	12.06	< 0.05
Inclination (º)	14.51	20.87	22.15	< 0.05
Volar Tilt (º) Articular	-9.80	5.77	5.94	< 0.05
Distance (mm)	-	5.73	4.60	< 0.05

Out of all patients, 94 (63.1%) reported being satisfied with the overall outcome at final follow-up, while 52 (34.9%) were not satisfied, and three (2.0%) did not provide a satisfaction response (Table 3). When comparing satisfaction outcomes, the mean AD at six weeks was slightly higher in the satisfied group (4.84 mm) compared to the unsatisfied group (4.50 mm), suggesting a potential association between greater preservation of articular height and improved patient-perceived outcomes.

**Table 3:** Patient Satisfaction

Satisfaction Status	Number of Patients	Percentage (%)
Satisfied	94	63.1
Not Satisfied	52	34.9
No Response	3	2

Mean postoperative AD for the satisfied group was 4.84 mm and for those not satisfied it was 4.50 mm. This suggests that higher AD values are associated with greater patient satisfaction.

## Discussion

Our findings demonstrate significant improvements in key radiological parameters (RH, RI, VT) following volar plate fixation of distal radius fractures. These results are consistent with prior studies showing that anatomical reduction correlates with better functional outcomes [5,6].

Although fixation initially restores the articular height (AD), a measurable collapse (mean: 1.13 mm) was observed at six weeks. This may be attributed to poor bone quality, early postoperative mobilisation, or insufficient subchondral support. Similar collapse patterns have been identified in older populations, especially with greater distances between distal screws and the subchondral plate [7,8].

Patient satisfaction was positively associated with higher postoperative AD values, suggesting that preservation of articular congruity plays a role in perceived outcomes. However, the association between radiological results and clinical satisfaction remains complex.

Recent work by Lee et al. aligns more closely with our findings, highlighting that older age and suboptimal screw placement contribute to height collapse, reinforcing the importance of intraoperative technique and bone quality in maintaining long-term stability [8].

Ng and McQueen emphasized that among various radiological parameters, only residual dorsal angulation significantly predicted poorer outcomes, while loss of radial length or inclination had a lesser impact [9]. In contrast, our study found significant changes across all three parameters (VT, RH, and RI), supporting a broader interpretation of their functional relevance.

Further, Schmidt et al. conducted a large prospective study (n = 366) and found a weak correlation between radiographic alignment and patient-reported outcomes after one year, challenging the assumption that better radiographs always yield better function [10]. While our study noted a trend linking AD collapse to patient dissatisfaction, we acknowledge that subjective outcomes are influenced by multiple variables, including pain, expectations, rehabilitation, and baseline function.

In line with our findings, Plant et al. questioned the assumption that better radiographs equate to better patient function. Their study found only a weak correlation between radiological and functional outcomes, especially beyond the early postoperative period, highlighting that fracture healing and recovery are multifactorial processes [11]. Similarly, our study suggests that radiographic improvement does not guarantee patient satisfaction, as seen in the slight differences in AD among those satisfied and unsatisfied.

While patient satisfaction appeared higher in those with greater postoperative AD values, the observed difference was small and not statistically tested. This should therefore be interpreted as a trend rather than a definitive association. Satisfaction in distal radius fracture management is influenced by multiple factors, including pain, rehabilitation quality, hand dominance, and occupational demands, which were not assessed in this study.

The strengths of this study include its relatively large sample size of 149 patients, which provides a robust dataset for assessing early outcomes of distal radius fixation. Radiological parameters were clearly defined and measured in a standardised way, allowing comparison with existing literature. All radiographs were independently reviewed by three consultant trauma and orthopaedic surgeons, which enhances the reliability of the findings. Importantly, by combining radiological outcomes with patient-reported satisfaction, the study reflects both objective and patient-centred perspectives, adding clinical relevance to the results.

However, limitations include its retrospective design, absence of validated functional scores such as Disabilities of the Arm, Shoulder, and Hand (DASH), and a short follow-up of six weeks. Our outcome measure of satisfaction, while useful, may oversimplify nuanced functional results. Additionally, variations in surgical technique and patient comorbidities (e.g., osteoporosis) were not controlled. Although radiographic measurements were independently reviewed by three consultants, no formal inter- or intra-observer reliability testing was performed. Finally, the statistical analysis was limited to paired t-tests, which were appropriate for assessing within-patient changes over time but did not allow for adjustment for potential confounders or exploration of independent predictors of satisfaction and collapse.

## Conclusions

Fixation of distal radius fractures with volar locking plates results in significant radiological improvement. Nevertheless, a mild articular collapse is evident by six weeks, and this decrease in AD may affect functional satisfaction. Efforts to optimise subchondral support and fixation technique may enhance both radiological and patient-reported outcomes.
